# Structural basis of Ornithine Decarboxylase inactivation and accelerated degradation by polyamine sensor Antizyme1

**DOI:** 10.1038/srep14738

**Published:** 2015-10-07

**Authors:** Donghui Wu, Hung Yi Kristal Kaan, Xiaoxia Zheng, Xuhua Tang, Yang He, Qianmin Vanessa Tan, Neng Zhang, Haiwei Song

**Affiliations:** 1 Institute of Molecular and Cell Biology, 61 Biopolis Drive, Singapore 138673; 2 Life Sciences Institute and Innovation Center for Cell Signaling Network, Zhejiang University, 388 Yuhangtang Road, Hangzhou 310058, China; 3 Department of Biochemistry, National University of Singapore, 14 Science Drive, Singapore 117543; 4 Shanghai Institute for Advanced Immunochemical Studies, ShanghaiTech University, 99 Haike Road, Pudong District, Shanghai, 201210, China

## Abstract

Ornithine decarboxylase (ODC) catalyzes the first and rate-limiting step of polyamine biosynthesis in humans. Polyamines are essential for cell proliferation and are implicated in cellular processes, ranging from DNA replication to apoptosis. Excessive accumulation of polyamines has a cytotoxic effect on cells and elevated level of ODC activity is associated with cancer development. To maintain normal cellular proliferation, regulation of polyamine synthesis is imposed by Antizyme1 (AZ1). The expression of AZ1 is induced by a ribosomal frameshifting mechanism in response to increased intracellular polyamines. AZ1 regulates polyamine homeostasis by inactivating ODC activity and enhancing its degradation. Here, we report the structure of human ODC in complex with N-terminally truncated AZ1 (cAZ1). The structure shows cAZ1 binding to ODC, which occludes the binding of a second molecule of ODC to form the active homodimer. Consequently, the substrate binding site is disrupted and ODC is inactivated. Structural comparison shows that the binding of cAZ1 to ODC causes a global conformational change of ODC and renders its C-terminal region flexible, therefore exposing this region for degradation by the 26S proteasome. Our structure provides the molecular basis for the inactivation of ODC by AZ1 and sheds light on how AZ1 promotes its degradation.

Ornithine decarboxylase (ODC) is an enzyme that catalyzes the first and rate-limiting step of polyamine biosynthesis in humans: the decarboxylation of ornithine to putrescine. ODC requires the co-factor pyridoxal 5′-phosphate (PLP) and is catalytically active only in the homodimer form^1^,^2^. Being ubiquitously expressed in mammals, bacteria and even parasites, ODC is indispensable because of its central role in polyamine biosynthesis. Disruption of its function by inhibitors leaves cells non-viable and causes embryonic lethality^3^,^4^. This is because the products of its catalysis – polyamines – play essential roles in normal cell growth and differentiation^1^.

Polyamines (putrescine, spermidine and spermine) are small aliphatic molecules implicated in a myriad of cellular processes, including DNA condensation, replication, RNA transcription, translation, ion channel function, embryonic development, angiogenesis, and apoptosis^5^,^6^,^7^,^8^,^9^,^10^. Given its essential role in normal cell proliferation, the depletion of polyamines and the inhibition of polyamine synthesis lead to growth cessation and consequently cell death^3^,^11^,^12^. Though polyamines are indispensible for cell viability, excessive accumulation of polyamines results in cytotoxicity of cells^13^,^14^. Hence, strict regulation of the levels of intracellular polyamines is required.

Polyamine homeostasis is achieved by Antizymes, a class of small proteins that inhibit ODC activity and polyamine uptake into cells^15^,^16^. There are at least three members in the human Antizyme family, namely, antizyme1 (AZ1), AZ2 and AZ3^17^. AZ1 was the first to be characterized and is the most prominent member of the family^18^. The expression of AZ1 is induced by a unique ribosomal frameshifting mechanism in response to increased levels of intracellular polyamines^17^,^19^,^20^. In turn, AZ1 inhibits polyamine uptake into the cells and stimulates excretion of polyamine out of the cell.

In addition, AZ1 has been shown to inhibit ODC activity and promote ODC degradation by the 26S proteasome^21^. Thus, a feedback loop is established: increased level of intracellular polyamines promotes the overexpression of AZ1, which inhibits ODC and promotes its degradation. Consequently, ODC catalysed polyamine synthesis is arrested. It is believed that AZ1 binding to ODC causes the catalytically active homodimer to dissociate. Consequently, the ODC active site, which is composed of residues at the interface of the homodimer, is disrupted and ODC becomes inactive. The formation of the ODC-AZ1 heterodimer is also thought to induce a conformational change that exposes the C-terminal region of ODC for the recognition and accelerated proteolysis by the 26S proteasome^22^. Unlike most proteins that are marked for degradation by ubiquitin conjugation, ODC is degraded by the 26S proteasome in an ubiquitin-independent manner^23^,^24^.

The regulation of ODC by AZ1 is crucial because elevated ODC activity has been observed in many epithelial cancers, such as colon, skin and prostate^1^, suggesting that ODC may function as a proto-oncoprotein^25^. As ODC activity is induced by various growth-promoting stimuli, such as growth factors, carcinogens and mitogens, the unregulated overexpression of ODC is readily linked to cellular transformation^25^ and leads to tumorigenesis^26^. Conversely, the expression of AZ1 inhibits ODC activity, exhibits anti-tumor activities and may be considered a tumor suppressor^17^,^27^.

Although ODC and AZ1 have been identified some 40 years ago^18^,^28^ and extensive studies have been carried out to examine the regulation of ODC by AZ1, two basic questions still remain to be clarified: how does AZ1 inactivate ODC and how does AZ1 stimulate the degradation of ODC by the 26S proteasome?

Here, we describe the crystal structure of human ODC-cAZ1 complex in the presence of co-factor PLP. Structural comparison of ODC-cAZ1 with the ODC homodimer reveals the molecular basis of ODC inactivation by AZ1 and provides insights into how AZ1 promotes proteasome-mediated degradation of ODC.

## Results

### Overall structure of ODC-cAZ1 complex

**Figure 1:**
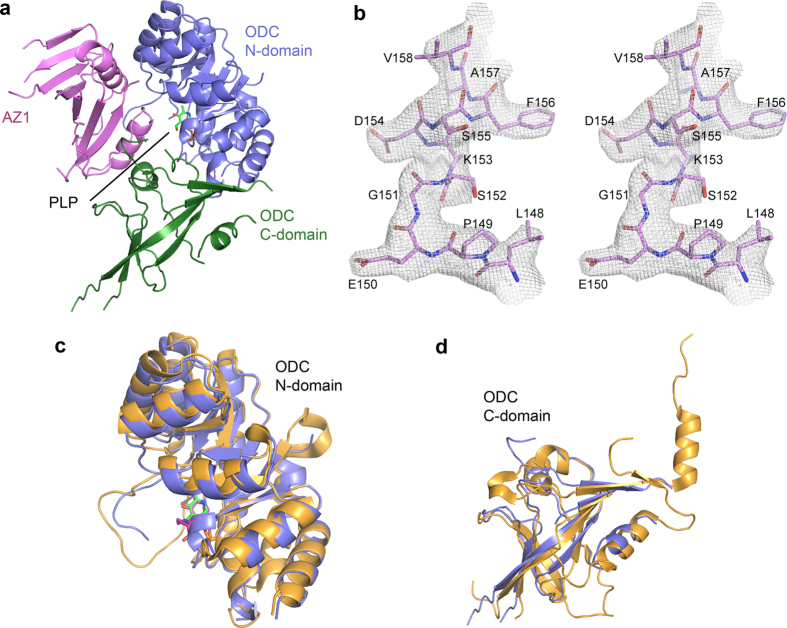
Crystal structure of ODC-cAZ1 complex. **(a**) ODC-cAZ1 complex forms a heterodimer, which consists of one ODC monomer and one AZ1 molecule (pink). AZ1 is embedded into a concave surface formed between the N- (purple) and C-domains (green) of ODC. PLP is shown as sticks (green). **(b)** Stereo image of the electron density map (2*Fo-Fc*; σ = 1.0) for a region of cAZ1 (pink, residues 148–158). **(c)** Overlay of N-terminal domain of ODC from our structure (purple) with that of human ODC dimer structure (yellow, PDB code: 1D7K) reveals no change in protein fold. PLP from ODC dimer structure is shown as sticks (magenta). **(d)** Overlay C-terminal domain of ODC from our structure (purple) with that of the human ODC dimer structure (yellow, PDB code: 1D7K) reveals almost no change in protein fold.

The crystal structure of ODC-cAZ1 complex was solved at a resolution of 3.2 Å (Fig. 1A). Electron density map for a region of cAZ1 (residues 148–158) is shown in Fig. 1B. Data collection and final refinement statistics are summarized in Table 1. In the asymmetric unit of the crystal structure, the ODC-cAZ1 complex forms a heterodimer, which consists of one ODC monomer and one cAZ1 molecule. The ODC monomer consists of two domains: the N-terminal TIM-like β/α-barrel domain (residues 44–283), which binds the co-factor, and the C-terminal domain (residues 19–43, 284–410). cAZ1, comprising a sheet of eight mixed parallel and anti-parallel strands and two helices^10^, is embedded in a concave surface formed between the two domains of ODC. Overlay of ODC N-terminal domains from our structure and the human ODC dimer structure (PDB code: 1D7K)^29^ gave an root-mean-square deviation (r.m.s.d.) of 0.75 Å, while overlay of the C-terminal domains gave an r.m.s.d. of 1.29 Å. (Fig. 1C,D). These results indicate that the binding of AZ1 does not alter protein fold of individual domains of ODC.

### Interactions between ODC and AZ1

**Figure 2:**
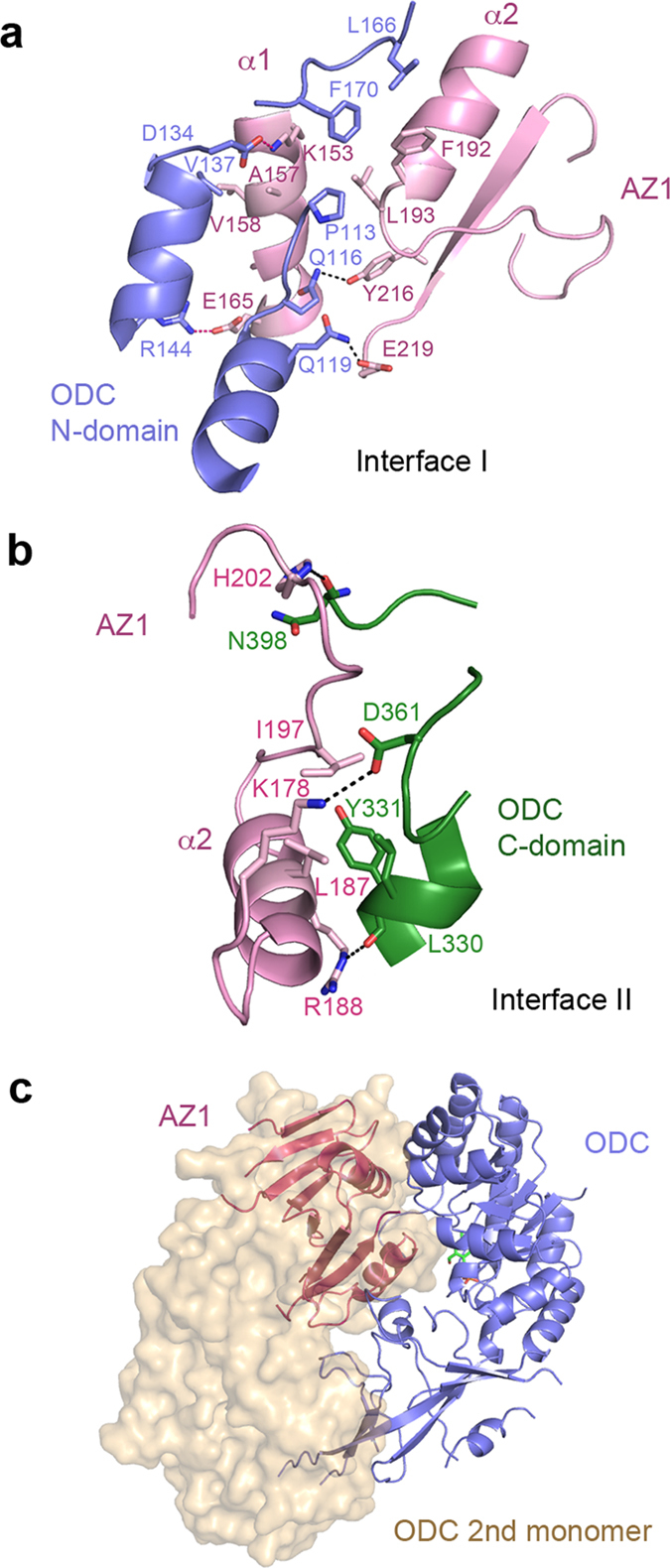
AZ1 binding to ODC monomer. **(a)** Residues involved in hydrogen bonds (black dash), ionic (pink dash) and hydrophobic interactions between ODC (purple) and AZ1 (pink) at interface I are shown as sticks. **(b)** Residues involved in the interactions between ODC C-domain (green) and AZ1 (pink) at interface II are shown as sticks. Color scheme is as before. **(c)** AZ1 (magenta) occupies the position of the C-terminal domain of the second ODC monomer (beige transparent surface) of the catalytically active homodimer.

The N- and C-domains of ODC interact with AZ1 to generate two interfaces, I and II, with buried solvent-accessible surfaces of ~2000 Å^2^ and ~1400 Å^2^ respectively. A network of hydrogen bonds, salt bridges and hydrophobic interactions mediates the binding of ODC to AZ1. At interface I (Fig. 2A), residues 113–170 of the N-terminal domain of ODC interact with helix α1, α2 and adjacent regions of AZ1. In particular, D134 and R144 of ODC form ionic interactions with K153 and E165 of AZ1 respectively. Two hydrogen bonds were also observed between Q116, Q119 of ODC and Y216, E219 of AZ1. Furthermore, the interaction at interface I is strengthened by two hydrophobic clusters: one cluster comprising V137 of ODC, A157 and V158 of AZ1 and the other cluster comprising P113, L166, F170 of ODC, F192 and L193 of AZ1.

At interface II (Fig. 2B), residues 300–398 of the C-terminal domain of ODC associate with helix α2 and adjacent regions of AZ1. Specifically, D361 of ODC forms an ionic interaction with K178 of AZ1. The carbonyl groups of Y331 and N398 of ODC form hydrogen bonds with the side-chains of R188 and H202 of AZ1 respectively. Additional contacts are mediated by hydrophobic interactions involving L330, Y331 of ODC, L187 and I197 of AZ1.

Consistent with the importance of residues at these two interfaces for ODC-AZ1 interaction, mutagenesis showed that replacing residues 117–140 of mouse ODC with the equivalent region of trypanosome ODC abolishes AZ1 binding^21^. In addition, substitutions of residues 119 and 137 of human ODC by His and Asp respectively reduce the binding of ODC to AZ1 substantially^30^. Moreover, mutations of K153, L193 and E219 of AZ1 to Ala reduce its binding to ODC by 3–8 folds^31^.

### AZ1 binding disrupts ODC substrate binding site

Overlay of our ODC-cAZ1 structure with the ODC homodimer structures shows AZ1 occupying the position of the C-terminal domain of the second ODC monomer^2^,^32^ (Fig. 2C). Therefore, the binding of AZ1 to ODC occludes the binding of a second molecule of ODC to form the active homodimer. In addition, the binding of AZ1 disrupts the substrate binding site of ODC, which is made up residues from both monomers of the active homodimer (D322 from one monomer, C360 and D361 from the second monomer)^33^,^34^.

The interface of the ODC homodimer contains two active sites for the ornithine decarboxylation reaction. Each active site is composed of one PLP binding site, made up mainly of the N-terminal domain of one monomer, and one substrate binding site, made up mainly of the C-terminal domain of the second monomer. As the binding of AZ1 occupies the position of the C-domain of ODC, it disrupts the substrate binding site, while leaving the co-factor binding site largely undisturbed.

### AZ1 binding induces global conformational change of ODC

**Figure 3:**
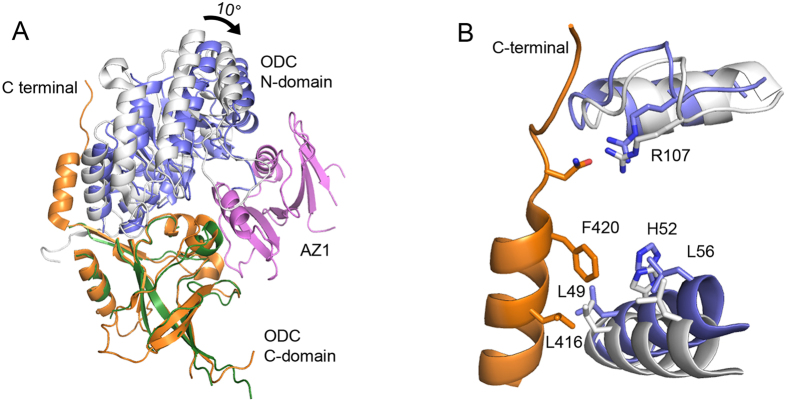
Binding of AZ to ODC induces global conformational change. **(a)** Overlay of ODC C-domain from our structure (green) with that of human ODC dimer structure (orange, PDB code: 1D7K) shows that that N-domain (purple) moves by about 10° towards AZ1, in the ODC-cAZ1 complex. **(b)** C-terminal helix (orange) of the ODC dimer structure is anchored mainly by hydrophobic interactions and a hydrogen bond with the N-domain (white). Binding of AZ1 causes the N-domain residues (purple) to be shifted away from the C-terminal helix.

Comparison of our ODC-cAZ1 structure with the ODC homodimer structure (PDB code: 1D7K)^29^ clearly shows that AZ1 binding induces a global conformational change of ODC. By overlaying the C-terminal sheet domain of ODC from both structures, we observed that the N-terminal domain of ODC moves by about 10° towards AZ1. The ODC molecule thus adopts a conformation whereby the angle between the N- and C-terminal domains is reduced (Fig. 3A). The same conformational change and degree of movement is also observed when we compared our structure with the ODC homodimer structures of other species, such as mouse and *Trypanosoma brucei* (PDB codes: 7ODC and 1NJJ)^2^,^35^.

Upon further analysis, we noticed another significant change brought about by the binding of AZ1. Based on all the available structures of human ODC homodimer (PDB codes: 1D7K, 2OO0 and 2ON3)^2^,^36^, the C-terminal tail of ODC (residues 424–461) has been shown to be disordered and non-visible, while the helix (residues 411–421) preceding this flexible region is clearly ordered. This helix is anchored mainly by hydrophobic interactions with a helix from the N-terminal domain (residues 45–58). The hydrophobic interactions involve residues L416 and F420 of the C-terminal helix and L49, H52 and L56 of the N-terminal helix. In addition, N422 of the C-terminal helix forms a hydrogen bond with R107 of the N-terminal domain (Fig. 3B). These interactions contribute to the stability and rigidity of the C-terminal helix.

In contrast, this ordered C-terminal helix becomes disordered and non-visible in our ODC-cAZ1 structure (Fig. 3A). Close examination showed that the residues from the N-terminal domain, which contacts the C-terminal helix, are shifted away by approximately 2.6 Å in response to AZ1 binding (Fig. 3B). Consequently, the C-terminal helix is released from the anchored position into the solvent-exposed area.

## Discussion

While previous biochemical and biophysical data by other groups have shown that AZ1 inactivates ODC by dissociating the ODC homodimer, we now have structural evidence to support this hypothesis. Our crystal structure undoubtedly shows the association of AZ1 with one monomer of ODC, which occludes the binding of a second ODC monomer to form the catalytically active ODC homodimer. This is in agreement with the sedimentation velocity analysis^37^, and other observations^21^,^38^,^39^, which show AZ1 and ODC exist as a heterodimer in solution. The structure of ODC homodimer showed that dimerization of human ODC buries a solvent-accessible surface of ~5050 Å^2^, which is ~1650 Å^2^ more than the buried surface upon formation of the ODC-AZ1 heterodimer^32^,^34^. Protein interface analysis using PDBePISA^40^ revealed that the ODC homodimer has 34 hydrogen bonds and 10 salt bridges at the interface, while ODC-AZ1 heterodimer has only 14 hydrogen bonds and 5 salt bridges. Given the substantial difference in buried surface and number of interactions at the interfaces, it seems energetically unfavorable for AZ1 to first break the ODC homodimer and then form the ODC-AZ1 heterodimer.

Nevertheless, ODC has been shown to undergo a rapid equilibrium between the active dimer and the inactive monomer *in vivo* and *in vitro*^39^,^41^,^42^,^43^. In addition, AZ1 is known to preferentially associate tightly with the inactive ODC monomer^39^. Therefore, depletion of the ODC monomer population by AZ1 binding may drive the rapid equilibrium towards the direction of ODC dissociation. Consequently, the binding of AZ1 dissociates ODC homodimer, which is the catalytically active form. Since the active site is composed of residues from both monomers, the binding of AZ1 disrupts the ODC dimer interface and renders ODC inactive.

To downregulate polyamine synthesis, AZ1 not only inactivates ODC, but also primes it for degradation. Unlike most proteins, which need to be ubiquitinated before proteolysis, ODC is one of the rare proteins that are degraded in a ubiquitin-independent manner by the 26S proteasome. AZ1 is not only essential for the degradation of ODC, it also shortens the half-life of ODC from the basal level of 1.5 hours to approximately 15 minutes^23^,^38^,^44^. The 37 residues at the C-terminus of ODC (amino acids 424–461) are believed to be the recognition site for the 26S proteasome^22^,^45^, as truncation of this region renders the protein more stable without affecting its activity^46^. This notion is supported by all the available human ODC homodimer structures, which show that these C-terminal residues are exposed to the solvent, highly flexible and mobile.

Li and Coffino have also shown experimentally that an accessible C-terminus of ODC is required for 26S proteasome recognition and degradation^22^. Either blocking the C-terminus of ODC with a specific antibody or tethering the C-terminus to the N-terminus prevented antizyme-promoted degradation of ODC. These results suggest that AZ binding to ODC exposes the C-terminus of ODC, making it more accessible for the recognition and degradation by 26S proteasome.

Our crystal structure provides additional support for this hypothesis by revealing the conformational changes of ODC upon AZ1 binding: the N-terminal domain of ODC moves by about 10° with respect to its C-terminal domain. This movement is accompanied by the transition of an ordered C-terminal helix (residues 411–421) to a disordered state, as the residues involved in stabilizing the helix have shifted by about 2.6 Å. With the steric restraints of the helix being lifted, these amino acids, together with the C-terminal 37 residues, now become highly flexible and solvent-exposed for more efficient proteasome recognition and degradation.

Given the important role of polyamines in cell growth and transformation, our crystal structure of ODC-cAZ1 heterodimer sheds light on polyamine homeostasis. As ODC is ubiquitously expressed in eukaryotes, fungi and parasites, ODC inhibitors have been shown to be useful in the treatment of various diseases, such as African sleeping sickness and Pneumocystis pneumonia in AIDS patients^47^,^48^. In addition, ODC is considered to be a proto-oncoprotein as the overexpression of ODC leads to tumorigenesis. Thus, inhibitors of ODC may be used as cancer chemotherapeutic or chemoprevention agents^49^. Difluoromethylornithine (DFMO) is an example of an ODC inhibitor that is undergoing clinical trials for use in cancer chemoprevention^50^. Our crystal structure, which shows ODC in an inactivated state bound to AZ1, will provide additional structural information for the design of novel ODC inhibitors.

## Methods

### Cloning, expression and purification

The genes encoding full length ODC and an N-terminal truncated AZ1 (residues 69–228, designated as cAZ1) from *Homo sapiens* were cloned into pGEX-6p-1 (GE Healthcare) to generate glutathione S-transferase (GST) fusion proteins with a PreScission protease (GE Healthcare) cleavage site. GST-tagged target protein was expressed in E. coli BL21 (DE3), grown at 37 °C in LB medium containing 100 μg/ml ampicillin. Cells were induced with 0.4 mM IPTG and grown at 18 °C for an additional 16 h prior to harvest. Cells were then sonicated in buffer A containing 20 mM Tris buffer (pH 8.5), 500 mM NaCl and 2 mM DTT. Cell debris was removed by centrifugation at 18,000 rpm at 4 °C. The supernatant containing GST-tagged ODC or cAZ1 was incubated with glutathione Sepharose 4B beads (GE Healthcare) pre-equilibrated in buffer A. The column was washed with excess buffer A, and protein was eluted with 20 mM reduced glutathione. The GST tag was cleaved with PreScission protease at 4 °C overnight. After desalting into buffer B, containing 20 mM Tris buffer (pH 8.5), 150 mM NaCl and 2 mM DTT, a second glutathione Sepharose column was used to remove the GST tag. The protein was further purified in Superdex-200 26/60 column (GE Healthcare) equilibrated in buffer B.

### Crystallization

The purified ODC and cAZ1 were mixed and incubated on ice for 1 hour with cAZ1 being in excess. Superdex-200 26/60 column, equilibrated in buffer B supplemented with 1 mM PLP (Sigma), was then used to purify ODC-cAZ1 complex. The purified ODC-cAZ1 complex was concentrated to 7.7 mg/ml. Hanging-drop vapor diffusion method was used to grow crystals at 15 °C. Cube-shaped crystals of ODC-cAZ1 complex were grown by mixing 1 μl of protein sample with 1 μl of reservoir solution (0.2 M di-Ammonium tartrate and 20% polyethylene glycol 3350). Crystals were transferred to cryoprotectant solution (0.2 M di-Ammonium tartrate, 25% polyethylene glycol 3350 and 15% Glycerol) in serial steps and frozen in liquid nitrogen

### Data Collection, Structure Determination and Refinement

The diffraction data of ODC-cAZ1 crystals was collected at beamline X06SA (PXI, SLS, Switzerland). The data was integrated with XDS^51^, merged and scaled with Scala from the CCP4 suite^52^. Using Phaser-MR in Phenix^53^ and a search model of human ODC monomer (PDB code: 1D7K, chain A), a clear solution for ODC was identified and the density for cAZ1 was partially visible. A human AZ1 model was generated from Swiss Model based on rat antizyme NMR structure (PDB code: 1ZO0), and fitted into the density. Iterative refinement was carried out using Phenix.refine^54^ and model building was carried out using COOT^55^. The model quality was checked with the program PROCHECK^56^. Figures were prepared using Pymol^57^.

## Additional Information

**Accession code**: The coordinates and structure-factor amplitudes of ODC-cAZ1 have been deposited to PDB with accession code 5BWA.

**How to cite this article**: Wu, D. *et al.* Structural basis of Ornithine Decarboxylase inactivation and accelerated degradation by polyamine sensor Antizyme1. *Sci. Rep.* **5**, 14738; doi: 10.1038/srep14738 (2015).

## Figures and Tables

**Table 1:** Crystallographic data and refinement statistics.

	ODC-cAZ1
Data collection statistics	
Space group	P2_1_2_1_2_1_
Unit cell dimensions a, b, c (Å)	53.2, 74.1, 157.1
Wavelength (Å)	1.0000
Resolution limit (Å)	42.7 −3.20 (3.36 −3.20)
Completeness (%)^a^	94.7 (94.4)
Rmerge (%)	8.4 (66.7)
*<I/σ(I)>*	15.7 (3.4)
Redundancy	10.0 (10.3)
Refinement statistics	
Resolution range (Å)	42.7 −3.2
Used reflections (N)	10122
*R*_*work*_*/R*_*free*_ (%)^b^	22.9/28.9
No. of atoms	3751
Protein/PLP	3735/16
r.m.s.d. Bond length (Å)/Bond angle (°)	0.006/1.254
Ramachandran plot (%)^c^	80.6/17.5/1.9/0.0

^a^Values in the highest resolution shell are shown in parentheses.

^b^*R*_*work *_= *Σ||Fobs| − |Fcalc||/Σ|Fobs|*. *R*_*free*_ is calculated identically with 5% of randomly chosen reflections omitted from the refinement.

^c^Fractions of residues in most favoured/allowed/generously allowed/disallowed regions of the Ramachandran plot were calculated using PROCHECK.
